# Prevalence of hypertension, diabetes, and associated risk factors in a rural community in Western Kenya: a population-based cross-sectional survey

**DOI:** 10.1186/s12889-026-28512-9

**Published:** 2026-07-13

**Authors:** Sylvester Ogutu, Jane Owenga, Amos Otedo, Dickens Omondi, Japheth Ogol, John Paul Bogers, Josefien van Olmen

**Affiliations:** 1 Department of Family Medicine and Population Health (FAMPOP), University of Antwerp, Wilrijk, Antwerp, Belgium; 2 School of Health Sciences, Jaramogi Oginga Odinga University of Science and Technology, Bondo, Kenya; 3 Laboratory of Cell Biology and Histology, University of Antwerp, Antwerp, Belgium; 4 Centre for Research in Health System Performance (CRiHSP), Yong Loo Lin School of Medicine, National University of Singapore, Singapore, Singapore; 5 Department of Family Medicine (DFM), National University of Singapore, Singapore, Singapore

**Keywords:** diabetes, hypertension, Kenya, prevalence, risk factors, rural

## Abstract

**Background:**

Hypertension and diabetes are rising causes of morbidity and mortality in sub-Saharan Africa, yet rural community-level data remain limited. National surveys often obscure local variation, hindering targeted prevention.

**Methods:**

We conducted a population-based cross-sectional study in Kakum-Kombewa, Siaya County, Kenya (Dec 2024-Jun 2025). Multistage sampling identified 816 households, yielding 1,046 adults aged ≥ 18 years. Participants underwent household screening for blood pressure, random blood glucose, and anthropometry. Individuals with screened positive values were referred to linked primary healthcare facilities for diagnostic confirmation. Prevalence was estimated using descriptive statistics, and associations were assessed with chi-square tests and generalized linear models.

**Results:**

The prevalence of known hypertension, diabetes, and comorbidity was 13.9%, 8.7%, and 4.9%, respectively. Diagnostic confirmation identified an additional 4.2% newly confirmed hypertension, 2.3% newly confirmed diabetes, and 2.5% newly confirmed comorbidity, yielding total prevalence estimates of 18.1% (95% CI: 15.8–20.6), 11.0% (95% CI: 9.2–13.1), and 7.4% (95% CI: 5.9–9.2). Risk factors included age ≥ 50 years, overweight/obesity, central adiposity, family history, and physical inactivity. Female sex, central adiposity, low activity, and family history remained significant predictors in multivariable models.

**Conclusion:**

This study reveals a substantial burden of both known and previously undiagnosed hypertension and diabetes in rural Western Kenya. Community-based screening, followed by referral and confirmatory testing, was effective in detecting new cases and identifying poor disease control among those with prior diagnoses. Strengthening routine NCD screening and linkage to primary care could improve early detection and management in similar rural settings.

**Supplementary Information:**

The online version contains supplementary material available at https://doi.org/10.1186/s12889-026-28512-9.

## Introduction

Hypertension and diabetes are the most significant non-communicable diseases (NCDs) due to their high prevalence, rising incidence, and contribution to mortality [1]. Together, they account for over 40% of NCDs worldwide [1–4], with diabetes increasing at the fastest rate [2, 3, 5]. Their coexistence more than doubles cardiovascular risk [6–9] and contributes to over 40% of NCD-related deaths globally [10]. In sub-Saharan Africa, hypertension affects 30–40% of adults, nearly two-thirds undiagnosed [11, 12], and one-third already living with complications [13, 14]. Diabetes prevalence is also rising rapidly, driven by urbanization, dietary shifts, sedentary lifestyles, and aging populations [3, 15].

Kenya mirrors this burden. National surveys reported 24% of adults with hypertension and 3% with raised fasting glucose [16]. Awareness remains low, with 73% of hypertensive individuals unaware of their diagnosis [17], and only 15.6% nationally aware of their condition. Rural data reveal even higher hidden burdens: in Nakuru, hypertension prevalence reached 50%, with over 60% undiagnosed [18]; in Busia County, western Kenya, diabetes prevalence was 5.3%, with more than half previously unknown [19]. These findings underscore a considerable hidden burden often obscured by national averages.

Effective resource allocation requires understanding local variations. Reliable estimates of prevalence and undiagnosed rates remain limited [20]. National surveys show regional differences in hypertension prevalence, awareness, treatment, and control [17], ranging from 23 to 24% in the 2015 Stepwise survey to 29% in the 2022 KDHS [11, 21, 22] and 33.2% in 2025 [1]. Multi-country analyses confirm that estimates vary by methodology and locality, highlighting the need for context-specific data [23]. Yet few community-based studies in Western Kenya provide combined, up-to-date estimates of hypertension and diabetes, their undiagnosed proportion, and associated risk factors [24]. Without such granular data, county-level and national planning risk misalignment with actual needs.

This study therefore aims to generate community-level evidence on the prevalence, undiagnosed burden, and risk factors for hypertension and diabetes in rural Western Kenya. Specifically, the objectives are to: (i) determine the prevalence of known hypertension and diabetes in Siaya County; (ii) identify the proportion of previously undiagnosed cases; and (iii) examine key risk factors and their association with hypertension, diabetes, and comorbidity in this population.

## Methods

### Study design and setting

This was a population-based cross-sectional study employing a quantitative, descriptive design. The study was conducted between December 2024 and June 2025 in Kakum-Kombewa, a rural sub-location within Alego-Usonga Sub-County, Siaya County, western Kenya. The area has an estimated population of 5,600 residents across 1,335 households, with most individuals engaged in peasant farming, fishing, and informal trading. The population is predominantly of low socioeconomic status and faces limited access to healthcare services, health education, and preventive screening for non-communicable diseases (NCDs). Kakum-Kombewa has 11 villages served by 11 Community Health Workers (CHWs) and three primary health facilities (2 dispensaries and 1 health center).

According to Kenya’s health infrastructure norms and standards, each level of care has defined infrastructure and service delivery roles. Community Health Workers (CHWs) act as the first point of contact for community healthcare services. Each village is served by one CHW, who provides basic health education and health promotion interventions. Their role is to provide preventive care, health promotion, early detection, screening, referral, and adherence support. Dispensaries and health centres (Level 2 facilities), which provide basic outpatient, preventive, and promotive services, are staffed by nurses and Clinical Officers [21, 25, 26].

**Fig. 1:**
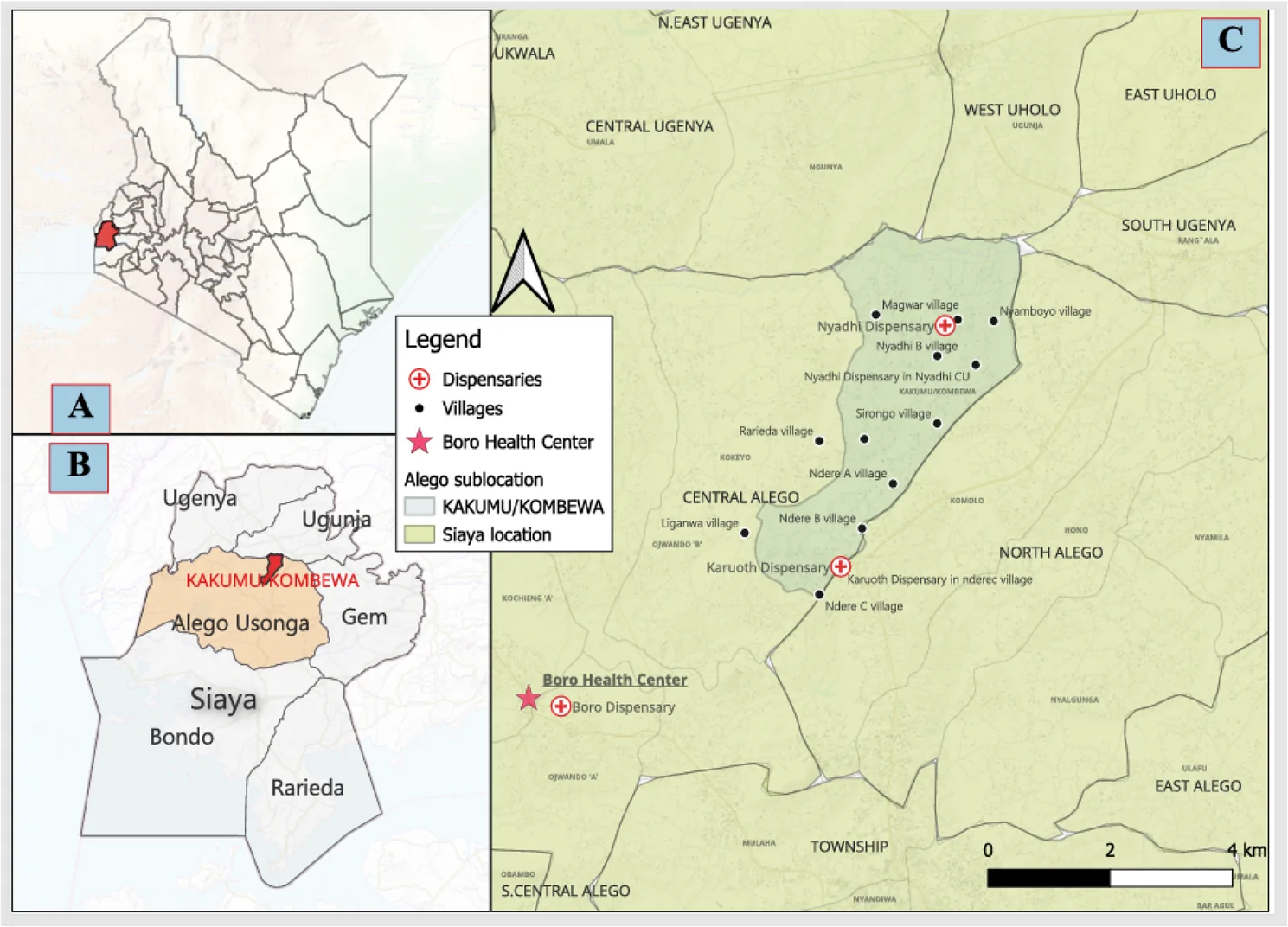
Map of the study area: Kakum-Kombewa Sub-location, Alego-Usonga Sub-County, Siaya County, Kenya

However, these facilities are few, widely dispersed, and in some cases not compliant with national norms and standards for service delivery [27]. As a result, access to diagnostic services for hypertension and diabetes is significantly constrained. For example, at the study site, the nearest government health facility equipped to screen for these non-communicable diseases (NCDs) is the Boro Health Centre, located approximately 15 km from Kakum-Kombewa (see Fig. 1).

This distance poses a considerable barrier for community members who require routine screening and regular follow-up care. Consequently, dispensaries staffed by nurses and Clinical Officers are underutilized in providing ongoing care or screening for individuals with, or at high risk of, hypertension and diabetes. These facilities often operate with limited diagnostic capacity, laboratory services, and drug supply, and in some cases do not fully comply with national service delivery standards [28].

### Target population

The target population included all adults aged 18 years and above residing in the selected households of Kakum-Kombewa Sub-location. Eligibility criteria included being a usual resident (lived in the household for ≥ 6 months), which is a requirement for one to be registered in the CHPs Community Register, willingness to provide informed consent, and not being acutely unwell or cognitively impaired in a way that would prevent meaningful participation at the time of the visit.

### Sample size estimation and sampling procedure

The sampling frame consisted of the 1,335 households in Kakum-Kombewa Sub-location, registered by Community Health Volunteers in the MOH 513 Household Register, which records household demographic data and is updated every six months. The sample size was calculated using the household survey formula:

#### Household sample size determination

Each household was considered a cluster within which eligible individuals would be recruited for the study. The minimum number of households required for the study was calculated using the approach described by Bennett et al. [10], implemented by the function epi.ssclus2estb within the R package “epiR”. The following parameters were used:

- Number of households available for sampling = 1,335.
- Average number of eligible individuals in each household = 2 (assuming an average household size of four, with two adults and two children) [29].
- Estimated prevalence of diabetes or hypertension = 200 per 1,000 population (i.e., 20%). This combined estimate reflects prior subnational studies in Kenya reporting hypertension prevalence ranging from 24% to 50% and diabetes from 3% to 5.3% [30].
- Desired margin of error (relative) = 0.1. For a 20% prevalence, a 10% relative margin implies ± 2% absolute error.
- Estimated intra-cluster correlation coefficient (ICC) = 0.1. This value is commonly used in community-based NCD studies in rural settings where moderate clustering is expected [31, 32].
- Confidence level = 0.95.

The minimum number of clusters (households) determined to be included was 518, corresponding to a minimum of 1,036 individuals. In total, 816 households with 1,084 participants were identified from the households, of which 1050 were eligible, but 4 withdrew after initially consenting to participate in the study, leaving a total of 1046 participants.

### Sampling procedure

Siaya County, one of Kenya’s six counties located within the Lake Victoria Basin, was purposively selected for this study due to its notably high disease burden. Specifically, Siaya ranks fourth in HIV prevalence and second in HIV incidence nationally, while non-communicable diseases account for nearly 40% of deaths and half of inpatient admissions, underscoring a dual burden (MOH). This study was nested within a larger study including Siaya, targeting six counties in Kenya under the School of Health Sciences of Jaramogi Oginga Odinga University of Science and Technology (JOOUST). The parent study is a sub-project in the institutional capacity-building project titled: ‘Strengthening Jaramogi Oginga Odinga University of Science and Technology Capacity in Natural Resource Management, Food Security and Health’ (Study number 001). To ensure a representative and systematic approach, a multistage sampling strategy was implemented, starting at the sub-county level and proceeding to individual households. At each administrative level, random selection methods were applied. Of the six sub-counties- Alego-Usonga, Bondo, Rarieda, Gem, Ugunja, and Ugenya- Alego-Usonga was randomly selected. Within Alego-Usonga, Central Alego Ward, Kakum-Kombewa sublocation was randomly selected for the study. All 11 villages within Kakum-Kombewa in Central Alego Ward were included in the sampling frame. Households in each village were identified through systematic random sampling. The households are listed by village in the MoH 513, Household Register. Village and household numbers in the Register were used to randomly select the first household, then every even household number was selected until the selected sample size for the village was reached. In practice, the first household in each village was the Community Health Worker’s (CHW) assigned household number, which served as the entry point into the community. This number is randomly assigned to each CHW in the village. The number of households selected per village was proportionate to its size (probability proportional to size-PPS) to ensure geographic representativeness across the sub-location. If a selected household was absent, the researcher made two return visits before recording it as non-response. Likewise, if eligible participants were unavailable, up to two follow-up visits were scheduled. Households or individuals who remained unavailable after repeated attempts were documented as non-responses, without substitution. The total number of households screened was 816, with a population of 1046 household members. All eligible household members who provided informed consent were enrolled in the study. Additional details are in Table 1.

**Table 1:** Actual proportionate distribution per village

Village	Number of Households	Household Population	Actual recruited households	% of enrolled Households
Liganwa	155	580	98	63
Ndere C	150	560	95	63
Sirongo	149	550	94	63
Nyamboyo	137	540	85	62
Uyore	135	460	84	62
Ndere B	116	562	71	61
Magwar	115	445	71	62
Nyadhi A	110	670	64	58
Ndere A	108	469	66	61
Nyadhi B	90	500	54	60
Rarieda	56	300	34	61
Total	1321		816	

### Data collection and procedures

Data were collected using structured interviews, anthropometric assessments, and biomedical measurements, all conducted at the household level by trained Community Health Workers. The data collection tools were adapted from the World Health Organization’s Stepwise Approach to NCD Surveillance (WHO Steps), IDF Diabetes Atlas, 2021, and pretested in a similar rural setting. The consenting participants underwent a structured interview that comprised demographics and risk factor assessment, Physical measurements (blood pressure, weight, height, waist circumference, and Key ratio measurements- waist-hip ratio, waist-height ratio), and capillary blood glucose testing. All data collection was conducted at the household level.

Sociodemographic and Behavioral Variables included age, sex, marital status, education, occupation, and geographical location; behavioral risk factors (smoking, alcohol use, dietary habits, and physical activity); and family history of hypertension or diabetes. Anthropometric and clinical measures comprised the assessment of weight and height measured using calibrated scales and stadiometers to calculate BMI. Waist circumference was measured at the midpoint between the lower rib and the iliac crest. Blood pressure was measured at one-minute intervals using an automated and validated Omron M2 digital blood pressure machine. If the 1st measurement was raised, the three measurements were taken, and the average of the three was taken if the. Participants rested quietly for five minutes before the first measurement. One-minute intervals separated subsequent readings on the same arm. Information on medication use was obtained through self-report by participants and verified, where possible, by checking prescription records or medication packaging brought by the participant. Participants with elevated blood pressure during community screening were referred to the nearest health facility, where confirmatory diagnostic testing was performed by trained clinicians and nurses using MoH standard diagnostic protocols for hypertension and diabetes (including repeat blood pressure measurement and fasting/random blood glucose testing).

Capillary blood glucose was measured using a calibrated On-Call Plus glucometer; fasting or random blood sugar measurements were recorded in mmol/l. For fasting assessment, participants were asked whether they had consumed anything other than plain water in the past 10 h (yes/no). If they answered “yes,” a random blood sugar test was conducted; if “no,” a fasting blood sugar test was performed. Table 2 below and Table 6 in Appendix 3 have more details. Further, see Fig. 3 in Appendix 2 for the risk score status for cardiovascular diseases.

**Table 2:** Objectives, variables, data collection, and references

Objective	Variables & Operational Definition / Measurement			How It Was Done (Data Collection)	Reference
1. To determine the prevalence of hypertension and diabetes, and their comorbidity	Blood pressure was measured three times on the left arm using an automated and clinically validated Omron M2 digital upperarm blood pressure monitor. Participants rested quietly for five minutes before the first measurement, and subsequent readings were taken at one minute intervals. The average of all three readings was documented for each participant if the 1st reading was elevated. Hypertension was defined as systolic blood pressure (SBP) ≥140 mmHg and/or diastolic blood pressure (DBP) ≥90 mmHg (new or undiagnosed cases). Pre-existing hypertension was confirmed by a health care worker or by evidence of current antihypertensive medication use. Other case definitions: • Known/pre-existing cases: Individuals who self-reported a prior diagnosis of hypertension or diabetes at the time of household screening. • Screening positive cases: Participants with elevated blood pressure or blood sugar detected during household screening. • Referred cases: Screening positive individuals referred to a primary healthcare facility for diagnostic confirmation. • Facility confirmed new cases: Participants without prior diagnosis who were confirmed with hypertension, diabetes, or both at the facility following referral. • Total burden: The sum of known/pre-existing cases and newly confirmed cases, representing the overall prevalence in the study population. • Comorbidity: Defined as individuals diagnosed with both hypertension and diabetes. These cases are reported separately but not double counted in single disease categories.			Blood Pressure was measured using a digital Omron M2 digital upper-arm blood pressure monitor, which was clinically validated by the Siaya County biomedical team.	WHO STEPS Manual (2020); IDF Diabetes Atlas (2021);
	Diabetes: Fasting glucose ≥7.0 mmol/L or random glucose ≥11.1 mmol/L; confirmed by HCW or current diabetes medication.			Blood glucose measured via On-Call Plus glucometer (fasting if ≥10 h since last meal; random if otherwise)	WHO STEPS Manual (2020); IDF Diabetes Atlas (2021);
	Diabetes/HTN status: Self-report of medication use for HTN/Diabetes and observed			Interview and direct confirmation of the availability of medicines • Medication verification: Information on medication use was obtained through self-report by participants and verified, where possible, by checking prescription records or medication packaging brought by the participant.	WHO STEPS Manual (2020); IDF Diabetes Atlas (2021)
	Comorbidity: Concurrent diagnosis of both HTN and Diabetes			Self-report of medication use and observed. • Medication verification: Information on medication use was obtained through self-report by participants and verified, where possible, by checking prescription records or medication packaging brought by the participant.	WHO STEPS Manual (2020); IDF Diabetes Atlas (2021)
2. To identify the prevalence of undiagnosed hypertension and diabetes	Undiagnosed HTN: SBP ≥140 mmHg and/or DBP ≥90 mmHg with no prior diagnosis or medication; confirmed on follow-up after referral.			Blood Pressure measured at screening; confirmation by HCW after referral • Confirmatory testing: Participants with elevated blood pressure during community screening were referred to the nearest health facility, where confirmatory diagnostic testing was performed by trained clinicians and nurses using MoH standard diagnostic protocols for hypertension and diabetes (including repeat blood pressure measurement).	WHO Global Monitoring Framework; IDF Diabetes Atlas (2021)
	Undiagnosed Diabetes: Fasting glucose ≥7.0 mmol/L or random glucose ≥11.1 mmol/L with no prior diagnosis or medication; confirmed by HCW after referral			Blood glucose measured via glucometer; Diagnostic confirmation by HCW after referral • Confirmatory testing: Participants with elevated blood pressure during community screening were referred to the nearest health facility, where confirmatory diagnostic testing was performed by trained clinicians and nurses using MoH standard diagnostic protocols for hypertension and diabetes (including repeat fasting/random blood glucose testing).	WHO Global Monitoring Framework; IDF Diabetes Atlas (2021)
	Cross-check with self-reported diagnosis and medication use.			Interview and observation • Medication verification: Information on medication use was obtained through self-report by participants and verified, where possible, by checking prescription records or medication packaging brought by the participant.	WHO Global Monitoring Framework
3. To explore the prevalence of key risk factors and their association with HTN and/or Diabetes	Age, Sex			Risk questionnaire (Part A)	WHO STEPS Survey Tool; WHO Global NCD Action Plan;
	Anthropometric measurements: Height, weight, waist, hip			Height and weight measured using tape and scale; waist/hip circumference measured with tape	WHO STEPS Survey Tool; WHO Global NCD Action Plan
	BMI categories: Normal (18.5–24.9), Overweight (25.0–29.9), Obesity I (30.0–34.9), Obesity II (35.0–39.9)			Calculated from measured height and weight	WHO STEPS Survey Tool
	Waist circumference risk: Men >102 cm = high risk; Women >88 cm = high risk Waist circumference			Measured with tape at standard anatomical sites	WHO STEPS Survey Tool
	Women	men	Risk Level		
	Less than 80 cm	Less than 94 cm	Normal		
	81–88 cm	94–102 cm	Moderate risk		
	More than 88	More than 102	High risk		
	Lifestyle and family history: Smoking, alcohol, physical activity, family history of HTN/Diabetes/heart disease			Questionnaire administered by trained CHWs	WHO STEPS Survey Tool; WHO Global NCD Action Plan
	Smoking status: Never, past, sometimes, daily			Self-report via questionnaire	WHO STEPS Survey Tool
	Alcohol use: Assessment of Alcohol Use: Alcohol consumption was assessed using a self-reported questionnaire item: “Do you consume alcohol daily?” Responses were coded as: 0 = No daily consumption 1 = Yes, daily consumption. Daily alcohol use was classified as an elevated risk factor for cardiovascular disease, consistent with established public health guidelines.			Self-report via questionnaire	WHO STEPS Survey Tool
	Physical activity: Assessment of Physical Activity: Physical activity was assessed using a self-reported questionnaire item that asked participants: “Do you undertake any physical activities for a minimum of 150 minutes in a week?” Responses were categorized as: 0 = sufficient activity (≥150 min of moderate physical activity per week) 1 = insufficient activity/Physical inactivity (<150 min of moderate physical activity per week). This classification was based on the World Health Organization (WHO) recommendations for the minimum weekly physical activity required for health benefits.			Self-report via questionnaire	WHO STEPS Survey Tool
	Family history: Parent/sibling with HTN/Diabetes/heart disease = positive			Self-report via questionnaire	WHO STEPS Survey Tool
	Waist-Height ratio This is a measure of abdominal fat distribution. Waist in (cm) /Height(cm)			Ratio is auto-calculated through REDCap	WHO
	Values	Risk Level			
	0.4- 0.49	Normal			
	0.5- 0.59	Moderate Risk			
	Above 0.6	High Risk			
	Waist-Hip Ratio: Ratio of waist(cm)/Hip(cm)			Auto-calculated through the REDCap	WHO
		women	Male		
	Normal	Less than=< 0.85 cm	Less than=< 0.90 cm		
	High risk	More than =>0.85 cm	More than =>0.90 cm		
	Risk score for cardiovascular diseases: Composite score from risk factors (age, sex, smoking, alcohol, BMI, physical activity, family history)			Derived from questionnaire responses	WHO STEPS Survey Tool

### Data management and analysis

Data were collected electronically using REDCap and exported to R statistical software (version 4.5.1) for cleaning, validation, and analysis. Descriptive statistics summarised participant demographics and the prevalence of diagnosed and undiagnosed hypertension and diabetes. Associations between primary outcomes and potential risk factors were examined using chi-square tests.

Prevalence estimates with 95% confidence intervals were calculated using standard methods (Wilson method via prop.test() in R). Standard logistic regression (generalized linear models with a binomial family and logit link) was applied to identify predictors of hypertension, diabetes, and comorbidity. The analysis did not incorporate clustering, household-level design weights, or probability proportional to size (PPS) adjustments in the regression models; accordingly, the prevalence estimates and regression findings presented here should be interpreted as descriptive of the study sample rather than formally weighted population estimates. The multistage sampling and PPS design were applied at the sampling stage to ensure geographic representativeness, as detailed in the sampling section.

Independent variables considered for inclusion in the logistic regression model were age, sex, smoking status, BMI, alcohol consumption, physical activity, and family history of NCDs. Variables were retained in the final model if they met the statistical significance threshold (*p* < 0.05) during model selection, following the purposeful selection approach described by Hosmer and Lemeshow [33], retaining predictors based on both statistical significance and clinical relevance. Sensitivity analyses were conducted to ensure robustness by using alternative model specifications and varying key assumptions to assess the consistency of the results.

The model-building process involved: (1) univariable screening of candidate predictors; (2) sequential multivariable modelling with stepwise addition of variables; and (3) evaluation of potential confounding effects. Final models reported adjusted odds ratios (AORs) with 95% confidence intervals and p-values.

Separate generalized linear models (GLMs) were built for five outcomes: (1) HTN and/or Diabetes combined; (2) HTN only; (3) Diabetes only; (4) comorbidity (HTN + Diabetes); and (5) previously undiagnosed status. Selected covariates included age, sex, BMI, smoking, alcohol use, physical activity, family history, waist/height measures, and blood pressure categories. For the chi-square analyses, disease status was defined as the presence of hypertension and/or diabetes, including comorbidity. All regression results are presented as odds ratios (ORs) with 95% confidence intervals and p-values; participants with missing data on any model covariate were excluded using complete case analysis (listwise deletion), and the number of complete cases included in each model is reported alongside the results. (see Tables 7, 8, 9 and 10, and 11 in the supplementary file for more details).

## Results

### Characteristics of study participants

Of the total 1,046 participants, females accounted for 732 (70.0%). Approximately half were aged 50 years or older (497; 47.5%), followed by those aged 30–49 years (402; 38.4%), while 147 (14.1%) were younger than 30 years. Overall, 255 (24%) were categorised into the high-risk score for cardiometabolic risk, and 791 (76%) into the low-risk category (Fig. 3 in the supplementary file).

**Fig. 2:**
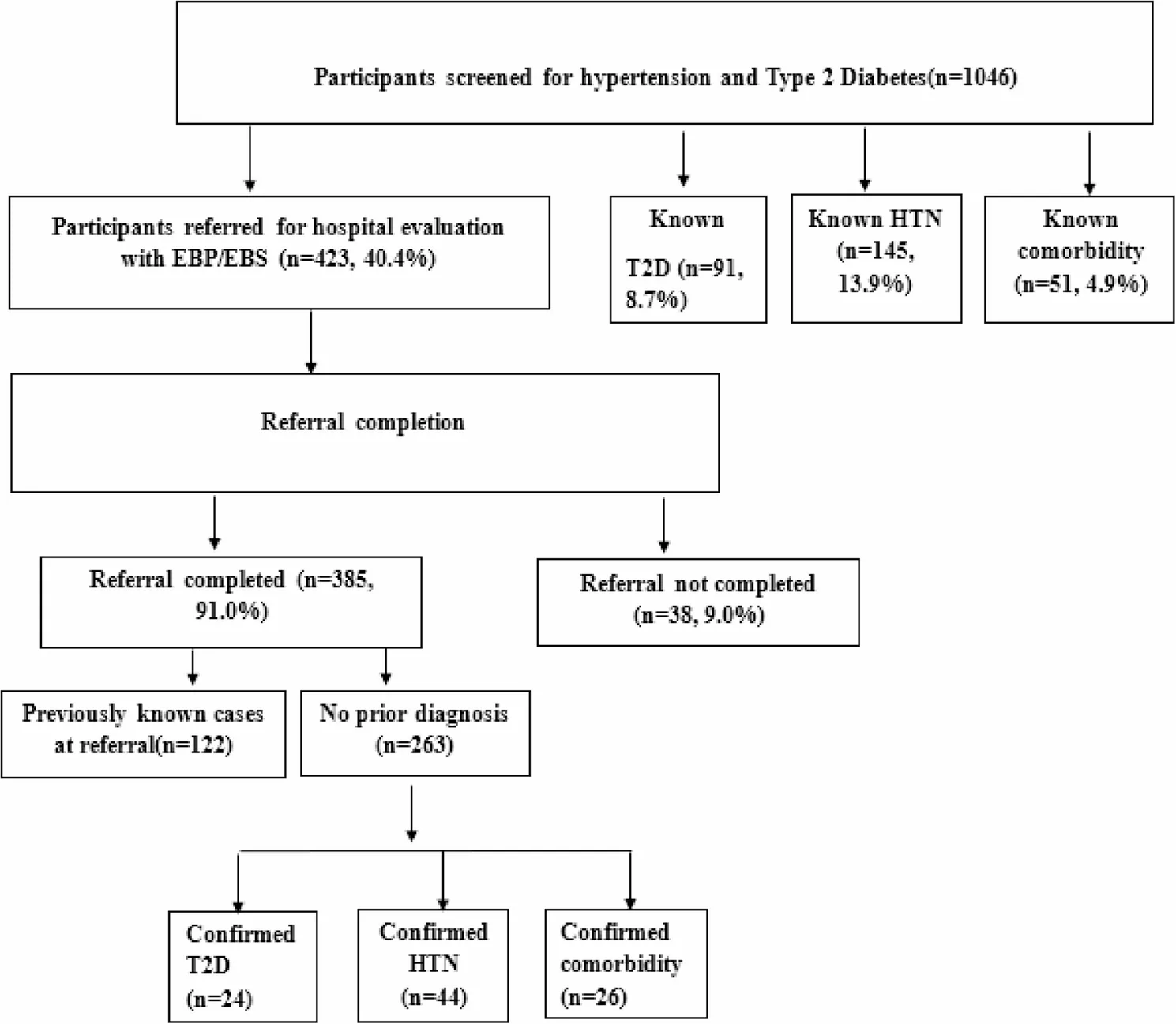
Participant flow diagram. key: *HTN* Hypertension, *EBP *Elevated Blood Pressure, *EBS* Elevated Blood Sugar, *T2D *Type 2 Diabetes

Figure 2 shows the participants' flow diagram through the study from screening to referral completion and diagnostic outcome.

### Prevalence of hypertension, diabetes, and comorbidity

Among the 1,046 adults screened, a total of 145 (13.9%) individuals were previously diagnosed with hypertension, and an additional 44 (4.2%) were newly confirmed, yielding an overall prevalence of 18.1% (95% CI: 15.8–20.6). For diabetes, 91 (8.7%) participants were known cases, while 24 (2.3%) were newly confirmed, resulting in a total prevalence of 11.0% (95% CI: 9.2–13.1). Comorbidity was observed in 51 (4.9%) individuals with prior diagnoses and 26 (2.5%) newly confirmed, giving a combined prevalence of 7.4% (95% CI: 5.9–9.2). These findings indicate that nearly one in five adults had hypertension, one in nine had diabetes, and one in thirteen lived with both conditions concurrently (Tables 3 and 4).

**Table 3:** Prevalence summary of hypertension, diabetes, and comorbidity

Condition	*N*	Known Cases *n* (%)	Newly Confirmed *n* (%)	Total Prevalence (95% CI)
Hypertension	1,046	145 (13.9%)	44 (4.2%)	18.1% (15.8–20.6)
Diabetes	1,046	91 (8.7%)	24 (2.3%)	11.0% (9.2–13.1)
Comorbidity (HTN + Diabetes)	1,046	51 (4.9%)	26 (2.5%)	7.4% (5.9–9.2)

**Table 4:** Prevalence of known cases of hypertension, diabetes, and comorbidity

Condition	Total *N*	Known Cases (*n*)	Prevalence (95% CI)
Hypertension	1,046	145	13.9% (11.8–16.1)
Diabetes	1,046	91	8.7% (7.1–10.6)
Comorbidity (HTN + Diabetes)	1,046	51	4.9% (3.7–6.4)

### Prevalence of previously undiagnosed cases

Out of 1,046 individuals screened, 423 (40.4%) were identified and referred to a health facility for further evaluation due to either elevated blood pressure (EBP) or elevated blood sugar (EBS), of whom 385 (91.0%) completed the referrals. Among those who completed referrals, 122 individuals were already diagnosed (known cases), while 263 had no prior diagnosis. Among those with no prior diagnosis, 24 individuals were confirmed with Diabetes, 44 with Hypertension, and 26 with comorbidity (both Diabetes and HTN) (Table 3).

### Key risk factors for hypertension and diabetes

Age was a notable risk factor: 42.3% of participants aged 50 years and older had HTN/Diabetes, compared with 29.9% among those under 30 years (*p* = 0.001). Anthropometric measures showed strong associations with HTN/Diabetes. Overall, 42.5% of participants were overweight or had mild to moderate obesity (BMI 25.0–39.9 kg/m²; *p* = 0.0002), and 67.9% of individuals classified as moderate to high-risk by waist–height ratio (*p* = 0.002) and 56.0% by waist circumference (*p* = 0.0322) presented with HTN and/or Diabetes. A family history of hypertension or diabetes (31.1%; *p* = 0.0005) and physical inactivity (< 150 min/week; *p* = 0.0031) were also significantly associated with HTN/Diabetes (Table 5).

**Table 5:** Participants’ characteristics and risk factors for HTN and/or diabetes status

Characteristics	Overall *N* = 1046 n (%)	HTN/Diabetes: No *n* (%)	HTN/Diabetes: Yes *n* (%)	*p*-value
Sex				
Male	314 (30.0)	190 (60.5)	124 (39.5)	0.201
Female	732 (70.0)	475 (64.9)	257 (35.1)	
Age Category				
Below 30 years	147 (14.1)	103 (70.1)	44 (29.9)	0.001***
30–49 years	402 (38.4)	275 (68.4)	127 (31.6)	
≥ 50 years	497 (47.5)	287 (57.7)	210 (42.3)	
BMI Category*				
Underweight	52 (5.1)	38 (73.1)	14 (26.9)	
Normal weight	508 (50.1)	339 (66.7)	169 (33.3)	
Overweight	279 (27.5)	183 (65.6)	96 (34.4)	
Mild Obesity	112 (11.0)	52 (46.4)	60 (53.6)	
Moderate Obesity	41 (4.0)	21 (51.2)	20 (48.8)	
Extreme Obesity	23 (2.3)	11 (47.8)	12 (52.2)	0.0002***
Waist-Hip Ratio				
Normal	379 (36.2)	247 (65.2)	132 (34.8)	
High risk	667 (63.8)	418 (62.7)	249 (37.3)	0.058
Waist Circumference				
Normal	460 (44.0)	307 (66.7)	153 (33.3)	
Moderate risk	248 (23.7)	162 (65.3)	86 (34.7)	
High risk	338 (32.3)	196 (58.0)	142 (42.0)	0.032*
Waist-Height Ratio*				
Normal	301 (32.1)	197 (65.4)	104 (34.6)	0.002**
Moderate Risk	424 (45.2)	279 (65.8)	145 (34.2)	
High Risk	213 (22.7)	111 (52.1)	102 (47.9)	
Family History				
No	721 (68.9)	484 (67.1)	237 (32.9)	0.001***
Yes	325 (31.1)	181 (55.7)	144 (44.3)	
Smoking				
Never	920 (88.0)	591 (64.2)	329 (35.8)	0.052
Past/Sometimes	108 (10.3)	61 (56.5)	47 (43.5)	
Daily	18 (1.7)	13 (72.2)	5 (27.8)	
Alcohol Use				
No	946 (90.4)	604 (63.8)	342 (36.2)	0.050
Yes	100 (9.6)	61 (61.0)	39 (39.0)	
Physical Activity				
< 150 min/week	252 (24.1)	140 (55.6)	112 (44.4)	
≥ 150 min/week	794 (75.9)	525 (66.1)	269 (33.9)	0.003**

*Missing data in Waist-height ratio(*n* = 108) and BMI(*n* = 31) were excluded from analysis. *p < 0.05, **p < 0.01, ***p < 0.001

### Multivariable GLM results

Table 6 presents the results of the generalized linear model (GLM) for the combined outcome of HTN and/or Diabetes (*n* = 1,046 complete cases). Odds ratios (ORs) are reported with 95% confidence intervals and p-values. The reference category for sex is Male; for physical activity, the reference is ‘at least 150 minutes per week’ (the recommended level); for family history, the reference is ‘No’.

**Table 6:** GLM-identified risk factors for hypertension and/or diabetes

Predictor	OR	95% CI	*p*-value	Significance
(Intercept)	1.33	0.36–5.00	0.668	
Age Category (ref: Below 30 years)				
Age 30–49 years	0.94	0.59–1.49	0.795	
Age ≥ 50 years	1.56	0.99–2.48	0.058	†
Sex (ref: Male)				
Female	0.58	0.39–0.86	0.007	**
Waist-Hip Ratio (ref: Normal)				
High Risk	1.07	0.79–1.52	0.397	
Waist Circumference (ref: Normal)				
Moderate risk	0.87	0.63–1.49	0.741	
High risk	0.61	0.38–1.11	0.053	†
Waist-Height Ratio (ref: Normal)				
Moderate Risk	0.84	0.53–1.71	0.641	
High Risk	0.73	0.41–1.36	0.149	
BMI (ref: Normal)				
Underweight	6.65	0.59–74.9	0.382	
Overweight	1.54	0.35–6.80	0.556	
Smoking (ref: Never)				
Past/Sometimes	1.08	0.67–1.75	0.743	
Daily	0.48	0.14–1.70	0.237	
Alcohol (ref: No)				
Yes	1.04	0.60–1.79	0.897	
Physical Activity (ref: ≥150 min/week)				
< 150 min/week (physically inactive)	1.81	1.32–2.47	0.001	**
Family History (ref: No)				
Yes	1.64	1.21–2.22	0.002	**

†Borderline significance (*p* < 0.10); *p < 0.05, **p < 0.01, ***p < 0.001. Reference categories: Sex = Male; Physical Activity = ≥ 150 min/week; Family History = No; Age = Below 30 years; BMI = Normal; Waist Circumference = Normal; Waist-Hip Ratio = Normal; Waist-Height Ratio = Normal. OR = Odds Ratio; CI = Confidence Interval. Complete cases *n* = 1,046

Two predictors were independently significant. Physical inactivity (less than 150 min of activity per week) was associated with significantly higher odds of HTN and/or Diabetes compared to meeting the recommended activity level (OR = 1.81, 95% CI: 1.32–2.47, *p* = 0.001). Family history of hypertension or diabetes was also significantly associated with increased odds (OR = 1.64, 95% CI: 1.21–2.22, *p* = 0.002). Female sex was associated with lower odds of HTN/Diabetes compared to males (OR = 0.58, 95% CI: 0.39–0.86, *p* = 0.007), noting that the reference category is male. Age ≥ 50 years showed a borderline association (OR = 1.56, 95% CI: 0.99–2.48, *p* = 0.058). No statistically significant associations were found for waist-hip ratio, waist circumference, waist-height ratio, BMI category, smoking, or alcohol use in the multivariable model.

## Discussion

### Prevalence of known and unknown cases of hypertension, diabetes, and comorbidity

This study assessed the prevalence of hypertension, diabetes, and their comorbidity in a rural population of Siaya County, Western Kenya, while also identifying previously undiagnosed cases and key risk factors associated with these conditions.

The results revealed that 18.1% of adults had hypertension, 11.0% had diabetes, and 7.4% were living with both conditions concurrently. These figures highlight the substantial burden of these conditions in this rural community. The prevalence of hypertension observed here is consistent with earlier rural Kenyan studies and findings within the region, where hypertension is consistently widespread within East Africa, reporting rates between 12% and 30% [34]. Diabetes prevalence is rising unevenly, with Tanzania showing a sharper increase (9.8%) compared to its neighbors (IDF 2024). The prevalence of diabetes in our study is broadly comparable to findings from other population-based surveys in sub-Saharan Africa, which report rates ranging from 3% to 6% in Kenya [35, 36] and 5% to 9.8% in Tanzania and Uganda [37–39], and 10.2% undiagnosed diabetes in Ethiopia. Importantly, our study also documented a high burden of previously unknown disease, where nearly one-third of participants not previously aware of any condition were referred for diagnostic confirmatory testing, and of these, 4.2% were confirmed hypertensive, 2.3% diabetic, and 2.5% had both conditions. Addressing both supply and demand factors simultaneously is a critical approach to achieving effective reductions in the burden of hypertension and diabetes in rural Kenya and other similar settings [40]. More evidence from Kenya highlights the need to strengthen health system capacity [41]. Furthermore, previous reports have demonstrated that financial protection mechanisms are essential to ensure equitable access and sustainability [42]. This study confirms well-established knowledge about risk factors for hypertension and diabetes in a rural Kenyan population [2, 3, 43–46]. Tailored interventions in rural settings should prioritize early detection, lifestyle modification, and regular risk assessment to reduce the burden of hypertension and diabetes.

### Strengths and limitations of the study

This study provides valuable community-level evidence on hypertension and diabetes in a rural Kenyan setting, using household screening linked to primary healthcare facilities for confirmatory testing. The large sample size and integration of community health workers enhance its relevance for rural NCD programs.

Several limitations should be noted. Referral completion was incomplete, potentially leading to underestimation of confirmed cases. Participation bias was possible given the predominance of women (70%), limiting generalizability to men. The cross-sectional design restricts causal inference, and reliance on single-visit screening may have introduced misclassification of blood pressure or glucose status. Behavioural risk factors were self-reported, raising the potential for recall or social desirability bias. The data are not fully representative of rural Western Kenya, as the focus was at the sub-county level; findings may therefore not be generalizable beyond this community, though they remain relevant for similar rural contexts. Participation bias may also have arisen because only two follow-up attempts were made for non-responders and absent participants. In regression analysis, standard complete-case methods (listwise deletion) were applied, excluding participants with missing covariates; the number of complete cases per model is reported in the Results. Finally, formal adjustment for complex survey design features (clustering, weighting, PPS) was not incorporated, so estimates reflect associations within the study sample and should be interpreted with this limitation in mind.

### Implications for research and practice

Our findings highlight the need to strengthen community-level screening and preventive programs for NCDs in rural populations. With community health workers (CHWs) now professionalized within Kenya’s health system, priority should be given to their training, supervision, and provision of affordable diagnostic tools. Community screening functioned not as a one-time event but as a continuum of care, linking detection with referral and follow-up. This approach underscores the value of standardized risk assessment guides and toolkits adapted to local outreach, supporting early identification and re-engagement of patients who may have disengaged from formal care [43, 47, 48].

Diagnosis alone does not ensure effective disease management. Among those previously diagnosed, 69% of hypertensive and 66.7% of comorbid participants required further clinical review following our household screening, revealing gaps in adherence, monitoring, and follow-up. Embedding household-level follow-up, led by CHWs and family caregivers, within the primary healthcare model is therefore essential for sustained disease control beyond health facilities.

A notable limitation of our study is the gender imbalance in the sample, with approximately 70% of participants being women. This disproportionate representation may influence the generalizability of findings, particularly in contexts where prevalence differs by gender. For example, regression estimates could be affected if gender-related differences in exposure or outcomes are not fully captured, potentially biasing effect sizes. While sensitivity analyses were conducted to test the robustness of results, future studies should aim for more balanced recruitment strategies to ensure that prevalence estimates and regression models more accurately reflect population-level distributions.

## Conclusion

Study demonstrates a substantial burden of both known and previously undiagnosed hypertension and diabetes in a rural western Kenyan community, underscoring the importance of community-based screening where NCD data remain limited. The detection of previously undiagnosed cases highlights the need for tailored interventions that prioritize early detection, regular risk assessment, expanded screening, and improved follow-up within primary health services. Among individuals with prior diagnoses, abnormal blood pressure and glucose values revealed challenges with disease control, pointing to the value of adherence support, lifestyle modification, and ongoing risk monitoring. Strengthening routine NCD screening and linkage to care could improve early detection and management in similar rural contexts.

## Supplementary Information

Supplementary Material 1.

## Data Availability

The datasets generated and analyzed during the current study are not publicly available due to institutional requirements. However, they can be accessed upon reasonable request through the corresponding author (SOO). Any data sharing will be subject to compliance with the approved ethical protocols and the principles governing research conduct at the University of Antwerp and within the VLIR scholarship framework.
