# Supplementary material for: Prevalence of hypertension, diabetes, and associated risk factors in a rural community in Western Kenya: a population-based cross-sectional survey
Source: BMC Public Health. 2026 Jul 13;26:2828. doi: 10.1186/s12889-026-28512-9 (PMC13644057; doi:10.1186/s12889-026-28512-9)
Supplement: Supplementary file 1 [file 12889_2026_28512_MOESM1_ESM.docx]

**Supplementary Material: Appendices**

**Prevalence and risk factors survey for hypertension and diabetes in rural western Kenya**

## Appendices

Appendix 1: **Questionnaire used by Community Health Promoters (CHPs) in rural Western Kenya**

Community-based Risk Assessment form for early Detection of Hypertension and Diabetes in Rural Western Kenya

**General Informational Information**

| Name of CHP: | Contact: |
| --- | --- |
| Name of village elder: | Contact: |
| Village: | Village code: |
| **Personal Details** |  |
| Name: | Any Identifier- DSSR2024-ID NO); |
| Age: | Social Health Insurance Schemes: (Y/N) ___________ |
| Sex: | Telephone No. |

Address (Nearest Landmark):

Marital Status:

**PART A: Risk Assessment**

Step 1.

Question	Range	Circle any/write Score

| 1. | What is your age? (in complete years) | Below 30 years |  | 0 |
| --- | --- | --- | --- | --- |
|  |  | 30-49 years |  | 1 |
|  |  | ≥ 50 years |  | 2 |
| 2. | Do you smoke or consume smokeless | Never |  | 0 |
| products? | | Used to consume in the past/ | | 1 |
|  |  |  |  |  |
|  |  | Sometimes now |  |  |
|  |  | Daily |  | 2 |
| 3. | Do you consume alcohol daily? | No |  | 0 |
|  |  | Yes |  | 1 |
| 4. | Measurement of waist (in cm) | Female | Male |  |
|  |  | 80 cm or less | 94 cm or less | 0 |
|  |  | 81-88 cm | 94-102 cm | 1 |
|  |  | More than 88 cm | More than 102 cm | 2 |
| 5. | Do you undertake any physical | At least 150 minutes in a week | | 0 |
| activities for minimum of 150 minutes in | | Less than 150 minutes in a week | | 1 |
| a week? | |  |  |  |
| 6. | Do you have a family history (anyone | No |  | 0 |
| of your parents or siblings) of high blood | | Yes |  | 2 |
| pressure, diabetes, and heart disease? | |  |  |  |

**Total Score (Risk Score)**

(A score above 4 indicates that the person may be at risk for these NCDs and needs to be prioritized for attending the routine NCD (HTN/T2D) awareness forum).

**PART B: Early Detection: Take the measurements and record appropriately**

**Step 2: ANTHROPOMETRIC MEASUREMENTS**

| Q. N | Description and Questions | Response |
| --- | --- | --- |
| Q1 | Height | Cm |
| Q2 | Weight | Kg |
| Q3 | Waist circumference | Cm |
| Q4 | Hip circumference | Cm |
| Q5 | Blood pressure, reading 1 | Systolic (mmHg)  Diastolic (mmHg) |
| Q6 | Blood pressure, reading 2 | Systolic (mmHg)  Diastolic (mmHg) |
| Q7 | Blood pressure, reading 3 | Systolic (mmHg)  Diastolic (mmHg) |

**Step 3: BIOMARKER MEASUREMENTS**

| Q. N | Description and Questions | Response |
| --- | --- | --- |
| Q8 | During the past 10 hours, have you had anything to eat or drink other than plain water? | 0 = No  1 = Yes |
| Q9 | Fasting Blood Glucose  -If not fasting, record as random blood sugar | mmol/l |
| Q10 | Today, have you taken insulin or other drugs(medication) that have been prescribed by a doctor or other health worker for raised blood glucose? | 0 = No  1 = Yes |
| Q11 | If not fasted, Record as Random blood sugar (MMOL/L) | mmol/l |

Q 12. Current prevalence of hypertension and diabetes in the community (Confirm from clinic books and/or by the presence of medications for hypertension and diabetes)

a) Are you already known and on hypertension drugs?

0 = No

1 = Yes

b) Are you already known and on diabetes drugs?

0 = No

1 = Yes

Q 13. Pain Score

| 1 | 2 | 3 | 4 | 5 | 6 | 7 | 8 | 9 | 10 |
| --- | --- | --- | --- | --- | --- | --- | --- | --- | --- |

**PART C: PHYSICAL EXAMINATION**

Q 14. General Exam

- Fair general appearance
- Sick looking
- Others; Specify

Q 15. Daily Participant Performance Status

- Normal Activity
- Symptomatic, ambulatory
- Debilitated, but bedridden (50% of the day)
- 100% Bedridden.

Q 16. Patient Status (following Screening)

- Referred
- Follow Up
- HBC
- Fair General Appearance
- Sick looking
- Others; Specify

Q 19. Form Status

- Incomplete
- Unverified
- Complete

**Appendix 2:**

|  |  |
| --- | --- |
| High Risk | 24.4% |
| Low Risk | 75.6% |

Figure 3: Risk score status for cardiovascular diseases

**Appendix 3:**

Table 7a: World Health Organization cut-off points and risk of metabolic complications: Indicator

Waist circumference

| Women | men | Risk Level |
| --- | --- | --- |
| Less than 80 cm | Less than 94cm | Normal |
| 81- 88cm | 94- 102 cm | Moderate risk |
| More than 88 | More than 102 | High risk |

Table 7 b: Waist-Hip Ratio

|  | women | Male |
| --- | --- | --- |
| Normal | Less than=< 0.85cm | Less than=< 0.90cm |
| High risk | More than =>0.85cm | More than =>0.90cm |
|  |  |  |

Table 7 c: Waist-Height ratio

This is a measure of abdominal fat distribution. Waist in (cm) /Height(cm)

| Values | Risk Level |
| --- | --- |
| 0.4- 0.49 | Normal |
| 0.5- 0.59 | Moderate Risk |
| Above 0.6 | High Risk |

Table 6 d: BMI Category

|  | BMI | class | Risk level |
| --- | --- | --- | --- |
| underweight | <18.5 |  |  |
| Normal | 18.5- 24.9 |  | Normal |
| Overweight | 25.0- 29.9 |  | Moderate risk |
| Obesity first | 30.0- 34.9 | 1(mild Obesity) | High risk |
| Obesity second | 35.0-39.9 | 2(moderate obesity) | Very high risk |
| Extreme obesity | >40.0 | 3(severe Obesity) | Extremely high risk |

Table 6 e: Classification of blood pressure (Kenya National Guideline for managing cardiovascular diseases, 2024)

| Category | Systolic |  | Diastolic |
| --- | --- | --- | --- |
| Optimal | < 120 | And | < 80 |
| Normal | 120- 129 | and/or | 80-84 |
| High normal | 130-139 | and/or | 85-89 |
| Grade 1 | 140- 159 | and/or | 90-99 |
| Grade 2 | 160-179 | and/or | 100-109 |
| Grade 3 | >= 180 | and/or | >= 100 |
| Isolated Systolic | >= 140 | And | < 90 |

Table 6 f: Blood Sugar Classification.

| Test | Normal | Pre-diabetes | Diabetes |
| --- | --- | --- | --- |
| Random Blood Sugar/ Glucose | 3.5- 7.8 mmol/l | 7.8- 11.0 mmol/l | > 11.1 mmol/l |
| Fasting blood sugar/glucose | 3.5- 5.6 mmol/l | 5.6- 6.9 mmol/l | > 7.0 mmol/l |

# **Appendix 4: Risk Factors and Their Association with HTN, Diabetes, Comorbidity, and Undiagnosed Status**

## ***Table 8: Risk Factors and Their Association with Hypertension (HTN)***

| **Predictor** | **OR** | **95% CI** | **p-value** |
| --- | --- | --- | --- |
| **Age** |  |  |  |
| Age 30–49 years | *0.319* | *0.109–0.935* | 0.037* |
| Age ≥50 years | *0.133* | *0.047–0.382* | <0.001*** |
| **Sex** |  |  |  |
| Female (vs Male) | *2.743* | *1.711–5.127* | <0.001*** |
| **Waist-Hip Ratio** |  |  |  |
| Normal vs High risk | *0.758* | *0.364–1.327* | 0.372 |
| **Waist Circumference** |  |  |  |
| Normal vs Moderate | *1.084* | *0.463–1.841* | 0.742 |
| Normal vs High risk | *3.647* | *1.521–8.432* | 0.004** |
| **Waist-Height Ratio** |  |  |  |
| Normal vs Moderate Risk | *1.493* | *0.491–3.411* | 0.467 |
| Normal vs High Risk | *1.692* | *0.731–2.633* | 0.715 |
| **BMI** |  |  |  |
| Normal vs Underweight | *6.475* | *0.910–6.961* | 0.062 |
| Normal vs Overweight | *1.285* | *0.615–2.686* | 0.503 |
| **Smoking** |  |  |  |
| Past/Sometimes vs Never | *0.883* | *0.484–1.612* | 0.686 |
| Daily vs Never | *NC** | *—* | 0.977 |
| **Alcohol** |  |  |  |
| Yes vs No | *0.947* | *0.453–1.984* | 0.886 |
| **Physical Activity** |  |  |  |
| <150 min/week vs ≥150 | *0.685* | *0.437–1.072* | 0.098 |
| **Family History** |  |  |  |
| Yes vs No | *0.461* | *0.306–0.696* | <0.001*** |

*Reference categories: Age = Below 30 years; Sex = Male; Waist-Hip Ratio = High risk; Waist Circumference = Normal; Waist-Height Ratio = Normal; BMI = Normal; Smoking = Never; Alcohol = No; Physical Activity = ≥150 min/week; Family History = No. NC* = not calculable due to model separation (extremely large SE). OR = Odds Ratio; CI = Confidence Interval. *p<0.05, **p<0.01, ***p<0.001.*

## **Table 9: Risk Factors and Their Association with Diabetes**

| **Predictor** | **OR** | **95% CI** | **p-value** |
| --- | --- | --- | --- |
| **Age** |  |  |  |
| Age 30–49 years | *1.452* | *0.509–4.144* | 0.486 |
| Age ≥50 years | *2.578* | *0.932–7.129* | 0.068 |
| **Sex** |  |  |  |
| Female (vs Male) | *0.473* | *0.225–0.995* | 0.048* |
| **Waist-Hip Ratio** |  |  |  |
| Normal vs High risk | *0.831* | *0.638–1.742* | 0.655 |
| **Waist Circumference** |  |  |  |
| Normal vs Moderate Risk | *0.317* | *0.543–1.662* | 0.511 |
| Normal vs High risk | *0.122* | *0.062–0.813* | 0.003** |
| **Waist-Height Ratio** |  |  |  |
| Normal vs Moderate Risk | *0.112* | *0.061–0.918* | 0.027* |
| Normal vs High Risk | *0.179* | *0.072–0.553* | <0.001*** |
| **Smoking** |  |  |  |
| Past/Sometimes vs Never | *0.717* | *0.305–1.686* | 0.447 |
| Daily vs Never | *NC** | *—* | 0.991 |
| **Alcohol** |  |  |  |
| Yes vs No | *0.666* | *0.202–2.196* | 0.504 |
| **Physical Activity** |  |  |  |
| <150 min/week vs ≥150 | *2.832* | *1.646–4.874* | <0.001*** |
| **Family History** |  |  |  |
| Yes vs No | *4.358* | *2.592–7.326* | <0.001*** |

*Reference categories as Table 7. Diabetes model excludes BMI (collinearity). NC* = not calculable due to model separation. *p<0.05, **p<0.01, ***p<0.001.*

## **Table 10: Risk Factors and Their Association with Comorbidity (HTN + Diabetes)**

| **Predictor** | **OR** | **95% CI** | **p-value** |
| --- | --- | --- | --- |
| **Age** |  |  |  |
| Age 30–49 years | *NC** | *—* | 0.985 |
| Age ≥50 years | *NC** | *—* | 0.985 |
| **Sex** |  |  |  |
| Female (vs Male) | *0.400* | *0.165–0.974* | 0.043* |
| **Waist-Hip Ratio** |  |  |  |
| Normal vs High risk | *0.816* | *0.370–1.798* | 0.614 |
| **Waist Circumference** |  |  |  |
| Normal vs Moderate | *0.724* | *0.411–2.543* | 0.641 |
| Normal vs High risk | *0.086* | *0.062–0.336* | 0.004** |
| **Waist-Height Ratio** |  |  |  |
| Normal vs Moderate Risk | *0.127* | *0.027–0.783* | 0.016* |
| Normal vs High Risk | *0.123* | *0.037–0.554* | 0.001** |
| **BMI** |  |  |  |
| Normal vs Underweight | *0.447* | *0.077–2.594* | 0.369 |
| Normal vs Overweight | *1.936* | *0.366–10.234* | 0.437 |
| **Smoking** |  |  |  |
| Past/Sometimes vs Never | *0.840* | *0.321–2.198* | 0.722 |
| Daily vs Never | *NC** | *—* | 0.994 |
| **Alcohol** |  |  |  |
| Yes vs No | *1.145* | *0.329–3.979* | 0.832 |
| **Physical Activity** |  |  |  |
| <150 min/week vs ≥150 | *0.864* | *0.410–1.820* | 0.701 |
| **Family History** |  |  |  |
| Yes vs No | *3.954* | *2.053–7.614* | <0.001*** |

*Reference categories as Table 7. NC* = not calculable due to model separation (quasi-complete separation for age categories). *p<0.05, **p<0.01, ***p<0.001.*

## **Table 11: Risk Factors and Their Association with Previously Undiagnosed HTN/Diabetes**

| **Predictor** | **OR** | **95% CI** | **p-value** |
| --- | --- | --- | --- |
| **Age** |  |  |  |
| Age 30–49 years | *0.608* | *0.276–1.337* | 0.216 |
| Age ≥50 years | *0.230* | *0.107–0.493* | <0.001*** |
| **Sex** |  |  |  |
| Female (vs Male) | *2.923* | *1.684–5.076* | <0.001*** |
| **Waist-Hip Ratio** |  |  |  |
| Normal vs High risk | *0.881* | *0.512–1.821* | 0.197 |
| **Waist Circumference** |  |  |  |
| Normal vs Moderate Risk | *1.271* | *0.451–2.169* | 0.432 |
| Normal vs High risk | *3.720* | *1.617–8.725* | 0.001** |
| **Waist-Height Ratio** |  |  |  |
| Normal vs Moderate Risk | *1.210* | *0.411–3.156* | 0.437 |
| Normal vs High Risk | *1.648* | *0.701–3.689* | 0.094 |
| **BMI** |  |  |  |
| Normal vs Underweight | *2.041* | *0.643–6.475* | 0.256 |
| Normal vs Overweight | *1.597* | *0.301–8.483* | 0.489 |
| **Smoking** |  |  |  |
| Past/Sometimes vs Never | *1.001* | *0.551–1.819* | 0.997 |
| Daily vs Never | *NC** | *—* | 0.976 |
| **Alcohol** |  |  |  |
| Yes vs No | *1.159* | *0.550–2.441* | 0.698 |
| **Physical Activity** |  |  |  |
| <150 min/week vs ≥150 | *0.388* | *0.257–0.587* | <0.001*** |
| **Family History** |  |  |  |
| Yes vs No | *0.352* | *0.239–0.520* | <0.001*** |

*Reference categories as Table 7. Outcome: previously undiagnosed HTN/Diabetes. NC* = not calculable due to model separation. *p<0.05, **p<0.01, ***p<0.001.*

**REFERENCES**

1.	Organization, W.H., Availability, price, and affordability of health technologies for the management of diabetes. 2025: World Health Organization.

2.	Organization, W.H., Assessing national capacity for the prevention and control of noncommunicable diseases: report of the 2021 global survey. 2023: World Health Organization.

3.	Ceriello, A. and S. Colagiuri, IDF global clinical practice recommendations for managing type 2 diabetes–2025. Diabetes Research and Clinical Practice, 2025: p. 112152.

4.	Romanello, M., et al., The 2023 report of the Lancet Countdown on health and climate change: the imperative for a health-centred response in a world facing irreversible harms. The Lancet, 2023. 402(10419): p. 2346-2394.

5.	Collaborators, G.L.B.P., Global, regional, and national burden of low back pain, 1990–2020, its attributable risk factors, and projections to 2050: a systematic analysis of the Global Burden of Disease Study 2021. The Lancet Rheumatology, 2023. 5(6): p. e316-e329.

6.	Collaboration, E.R.F., Diabetes mellitus, fasting blood glucose concentration, and risk of vascular disease: a collaborative meta-analysis of 102 prospective studies. The lancet, 2010. 375(9733): p. 2215-2222.

7.	Cosentino, F., et al., 2019 ESC Guidelines on diabetes, pre-diabetes, and cardiovascular diseases developed in collaboration with the EASD: The Task Force for diabetes, pre-diabetes, and cardiovascular diseases of the European Society of Cardiology (ESC) and the European Association for the Study of Diabetes (EASD). European heart journal, 2020. 41(2): p. 255-323.

8.	Murray, C.J., et al., Global burden of 87 risk factors in 204 countries and territories, 1990–2019: a systematic analysis for the Global Burden of Disease Study 2019. The lancet, 2020. 396(10258): p. 1223-1249.

9.	Zhou, B., Worldwide trends in diabetes since 1980: a pooled analysis of 751 population-based studies with 4.4 million participants. 2016.

10.	Bennett, J.E., et al., NCD Countdown 2030: worldwide trends in non-communicable disease mortality and progress towards Sustainable Development Goal target 3.4. The lancet, 2018. 392(10152): p. 1072-1088.

11.	Mohamed, S.F., et al., Prevalence of uncontrolled hypertension in people with comorbidities in sub-Saharan Africa: a systematic review and meta-analysis. BMJ open, 2021. 11(12): p. e045880.

12.	Ataklte, F., et al., Burden of undiagnosed hypertension in sub-saharan Africa: a systematic review and meta-analysis. Hypertension, 2015. 65(2): p. 291-298.

13.	Dzudie, A., et al., Prevalence, awareness, treatment and control of hypertension in a self-selected sub-Saharan African urban population: a cross-sectional study. BMJ open, 2012. 2(4): p. e001217.

14.	Adeloye, D., et al., Prevalence of dementia in Nigeria: a systematic review of the evidence. Journal of global health reports, 2019. 3: p. e2019014.

15.	Hossain, M.J., M. Al‐Mamun, and M.R. Islam, Diabetes mellitus, the fastest growing global public health concern: Early detection should be focused. Health Science Reports, 2024. 7(3): p. e2004.

16.	Okube, O.T., S. Kimani, and W. Mirie, Community-based lifestyle intervention improves metabolic syndrome and related markers among Kenyan adults. Journal of Diabetes & Metabolic Disorders, 2022. 21(1): p. 607-621.

17.	Mohamed, S.F., et al., Prevalence, awareness, treatment and control of hypertension and their determinants: results from a national survey in Kenya. BMC public health, 2018. 18(Suppl 3): p. 1219.

18.	Magara, G.M., Prevalence of Uncontrolled Hypertension and Associated Factors among Hypertensive Patients Attending Medical Outpatient Clinic, Thika Level 5 Hospital, Kiambu County, Kenya. 2022, JKUAT-COHES.

19.	Sarah, K., et al., Prevalence and risk factors associated with diabetes in Meru County, Kenya: a cross-sectional study. International Journal of Diabetes in Developing Countries, 2021. 41(3): p. 412-418.

20.	Arokiasamy, P., S. Salvi, and Y. Selvamani, Global burden of diabetes mellitus, in Handbook of global health. 2021, Springer. p. 1-44.

21.	Dibie, R. and F. Mainah, HEALTH POLICY AND CHALLENGES IN KENYA. Transforming Healthcare in Africa: p. 139.

22.	Knbs, I., Kenya demographic and health survey 2022. Nairobi, Kenya, and Rockville, Maryland, USA: KNBS and ICF, 2023.

23.	Irazola, V.E., et al., Hypertension prevalence, awareness, treatment, and control in selected LMIC communities: results from the NHLBI/UHG Network of centers of excellence for chronic diseases. Global heart, 2016. 11(1): p. 47-59.

24.	Mengesha, E.W., et al., Effectiveness of community-based interventions for prevention and control of hypertension in sub-Saharan Africa: A systematic review. PLOS Global Public Health, 2024. 4(7): p. e0003459.

25.	Mberu, B., et al., Landscape analysis of the Kenyan policy on the treatment and prevention of diarrheal disease among under-5 children. BMJ open, 2024. 14(8): p. e081906.

26.	Kalebi, A., Kenya and Covid-19, in Covid-19 and Governance. 2021, Routledge. p. 255-263.

27.	Health, M.o., Kenya Harmonized Health Facility Assessment (KHFA), 2018/2019. Nairobi: Ministry of Health, 2020.

28.	Patel, V., Clinical Trials in Kenya. 2006: SOMO Amsterdam.

29.	Statistics, K., The 2009 Kenya population and housing census. Nairobi: Kenya National Bureau of Statistics, 2019.

30.	Magliano, D.J. and E.J. Boyko, IDF diabetes atlas. 2021.

31.	Ouyang, Y., et al., Estimating intra-cluster correlation coefficients for planning longitudinal cluster randomized trials: a tutorial. International Journal of Epidemiology, 2023. 52(5): p. 1634-1647.

32.	Eldridge, S.M., O.C. Ukoumunne, and J.B. Carlin, The intra‐cluster correlation coefficient in cluster randomized trials: a review of definitions. International Statistical Review, 2009. 77(3): p. 378-394.

33.	Hosmer, D.W., S. Lemeshow, and R.X. Sturdivant, Applied logistic regression. 2000: Wiley New York.

34.	Shah, J., et al., Prevalence and risk factors of hypertension among clients seeking care at Selected Healthcare Facilities in Kenya. Plos one, 2025. 20(10): p. e0334255.

35.	Ayah, R., et al., A population-based survey of prevalence of diabetes and correlates in an urban slum community in Nairobi, Kenya. BMC public health, 2013. 13(1): p. 371.

36.	Pastakia, S.D., et al., Diabetes in sub-Saharan Africa–from policy to practice to progress: targeting the existing gaps for future care for diabetes. Diabetes, metabolic syndrome and obesity: targets and therapy, 2017: p. 247-263.

37.	Kavishe, B., et al., High prevalence of hypertension and of risk factors for non-communicable diseases (NCDs): a population based cross-sectional survey of NCDS and HIV infection in Northwestern Tanzania and Southern Uganda. BMC medicine, 2015. 13(1): p. 126.

38.	Guwatudde, D., et al., The epidemiology of hypertension in Uganda: findings from the national non-communicable diseases risk factor survey. PloS one, 2015. 10(9): p. e0138991.

39.	Bantie, G.M., et al., Prevalence of undiagnosed diabetes mellitus and associated factors among adult residents of Bahir Dar city, northwest Ethiopia: a community-based cross-sectional study. BMJ open, 2019. 9(10): p. e030158.

40.	Ouma OJ, Omondi D, Museve E, Owenga J, Bogers J, Abrams S, van Olmen J. Assessing the capacity of primary healthcare facilities and healthcare workers in managing diabetes and hypertension in Kisumu county, Kenya. BMC Health Serv Res. 2025 Sep 30;25(1):1221. doi: 10.1186/s12913-025-13390-5. PMID: 41029377; PMCID: PMC12482475.

41.	Bloomfield, H.E., et al., Deprescribing for community-dwelling older adults: a systematic review and meta-analysis. Journal of General Internal Medicine, 2020. 35(11): p. 3323-3332.

42.	Ogungbe, O., et al., Improving hypertension control in Nigeria: early policy implications from the Hypertension Treatment in Nigeria program. Global Health Research and Policy, 2024. 9(1): p. 26.

43.	Yazdi-Feyzabadi, V., M.H. Mehrolhassani, and A. Darvishi, Measuring catastrophic health expenditures and its inequality: evidence from Iran’s Health Transformation Program. Health policy and planning, 2019. 34(4): p. 316-325.

44.	Flor, L.S., et al., Community-based interventions for detection and management of diabetes and hypertension in underserved communities: a mixed-methods evaluation in Brazil, India, South Africa, and the USA. BMJ Global Health, 2020. 5(6): p. e001959.

45.	Chikwati, R.P., et al., Incident type 2 diabetes and its risk factors in men and women aged 40–60 years from four sub-Saharan African countries: results from the AWI-Gen study. The Lancet Global Health, 2025. 13(3): p. e459-e466.

46.	Wafeu, G.S., et al., Prevalence and associated factors of active smoking among individuals living with hypertension and/or diabetes in Africa: a systematic review and meta-analysis protocol. BMJ open, 2017. 7(10): p. e015444.

47.	Brauer, M., et al., Global burden and strength of evidence for 88 risk factors in 204 countries and 811 subnational locations, 1990–2021: a systematic analysis for the Global Burden of Disease Study 2021. The Lancet, 2024. 403(10440): p. 2162-2203.

48.	Organization, W.H., 2021 WHO health and climate change global survey report. 2021: World Health Organization.
